# Dosage and Dose Schedule Screening of Drug Combinations in Agent-Based Models Reveals Hidden Synergies

**DOI:** 10.3389/fphys.2015.00398

**Published:** 2016-01-06

**Authors:** Lisa C. Barros de Andrade e Sousa, Clemens Kühn, Katarzyna M. Tyc, Edda Klipp

**Affiliations:** ^1^Theoretische Biophysik, Humboldt-Universität zu BerlinBerlin, Germany; ^2^RNA Bioinformatics, Max Planck Institute for Molecular GeneticsBerlin, Germany

**Keywords:** agent-based models, drug treatments, fungal infections, *C. albicans*, host-pathogen interactions

## Abstract

The fungus *Candida albicans* is the most common causative agent of human fungal infections and better drugs or drug combination strategies are urgently needed. Here, we present an agent-based model of the interplay of *C. albicans* with the host immune system and with the microflora of the host. We took into account the morphological change of *C. albicans* from the yeast to hyphae form and its dynamics during infection. The model allowed us to follow the dynamics of fungal growth and morphology, of the immune cells and of microflora in different perturbing situations. We specifically focused on the consequences of microflora reduction following antibiotic treatment. Using the agent-based model, different drug types have been tested for their effectiveness, namely drugs that inhibit cell division and drugs that constrain the yeast-to-hyphae transition. Applied individually, the division drug turned out to successfully decrease hyphae while the transition drug leads to a burst in hyphae after the end of the treatment. To evaluate the effect of different drug combinations, doses, and schedules, we introduced a measure for the return to a healthy state, the infection score. Using this measure, we found that the addition of a transition drug to a division drug treatment can improve the treatment reliability while minimizing treatment duration and drug dosage. In this work we present a theoretical study. Although our model has not been calibrated to quantitative experimental data, the technique of computationally identifying synergistic treatment combinations in an agent based model exemplifies the importance of computational techniques in translational research.

## Introduction

Microbial pathogens are becoming increasingly resistant to existing antibiotics (WHO, 2014), leading to an urgent need for novel drugs, and an improvement of treatment strategies. Treatment of newly acquired infections in already hospitalized patients often enforces simultaneous administration of different drugs. In order to reduce potential deleterious side effects, treatment strategies must be carefully evaluated. In this study, we focused on the fungus *Candida albicans*, the most common causative agent of human fungal infections (CDC, 2014). In order to assess drug effects on a progress of fungal infections, we developed an agent-based model that allowed us to investigate *C. albicans* interactions with its host. We used this model to test combinatorial drug treatments as a conceptual cost efficient way to generate novel therapeutic strategies from existing drugs. Specifically, we systematically evaluated drug combinations that target multiple virulence aspects of the pathogen. We report synergistic drug effects and an unexpected stabilization of a theoretical medication upon specific treatment combinations.

*C. albicans* is commonly found in the human microflora without causing any harm to its host. Alterations in either the host immune system or the balance of the surrounding microflora can stimulate fungal overgrowth and colonization of epithelial surfaces by *C. albicans*. In turn, uncontrolled fungal overgrowth can result in an invasion of epithelial surfaces by *C. albicans* and dissemination of the fungus to internal organs causing systemic candidiasis—often fatal to the host (Perlroth et al., 2007). An important virulence factor of the fungus is its ability to switch between two morphological forms—the yeast and hyphal form. This morphological transition is often considered as essential for *C. albicans* pathogenicity (Lo et al., 1997). This specific virulence factor has been proposed as a potential drug target to fight the fungus (Jacobsen et al., 2012). Since the yeast-to-hyphae transition can be blocked by exogenously supplied farnesol it has also been proposed that farnesol may be used as a potential *C. albicans*-specific drug (Décanis et al., 2011). Also, it has been shown that hyphae are able to breach epithelial barriers (Phan et al., 2000), and a return to the yeast state is considered as necessary for the fungal dissemination within the bloodstream (Saville et al., 2003). To generate an appropriate immune response to the different states of *C. albicans* during colonization, it is critical for the host to distinguish between yeast and hyphal cells. Experimental results suggest that epithelial cells are in fact able to recognize the hyphal form and initiate an appropriate response tuned to the overall hyphal burden (Moyes et al., 2010). The hyphae-induced danger response activates a protective immune response, resulting in the release of a set of pro-inflammatory cytokines like IL-1α, IL-1β, IL-6, or TNF-α and chemokines such as IL-8 that act as chemoattractants and activators of host phagocytic cells, such as macrophages and polymorphonuclear neutrophils (PMNs) (Naglik and Moyes, 2011; Cheng et al., 2012). PMNs are activated by cytokines such as IL-22 (Ouyang et al., 2008), produced during *C. albicans* hyphae invasion by T helper cells 17, activated in turn by specific cytokines: IL-23, IL-1, IL-6 (Acosta-Rodriguez et al., 2007). While *C. albicans* cells were shown to be able to escape a macrophage attack (Ibata-Ombetta et al., 2001; Lorenz et al., 2004; Wellington et al., 2014), PMNs are considered to be efficient in killing *C. albicans* hyphal cells (Wozniok et al., 2008). Host, pathogen and commensal microflora all interact simultaneously to form a very dynamic environment. Which interactions within this environment favor or suppress the development of candidemia is impossible to resolve without formal analysis of the system. Here, we present an agent-based model (ABM) of *C. albicans* infection that describes the initial stages of the fungal invasion due to a disrupted microfloral balance. The main components of ABMs are discrete autonomous agents that interact with each other or their environment at discrete model time steps (*ts*) according to a set of rules (Chiacchio et al., 2014). We used the computational tool NetLogo (Wilensky, 1999) to stochastically simulate a 2-dimensional ABM. Extending previous considerations (Tyc and Klipp, 2011), we also implemented and analyzed the associated immune responses by the host. The model contained *C. albicans* agents that could switch between yeast and hyphal forms embedded in the epithelial environment that also comprised an actively growing microflora. We also included *C. albicans*-dependent cytokine release that in turn induced PMNs and macrophages recruitment to combat the pathogen. The model permitted to explore the potential of different treatment strategies *in silico* by systematically screening different combinations of a hypothetical farnesol-derived drug that inhibits the yeast to hyphae transition as well as an antifungal drug to determine optimal treatment strategies.

## Materials and methods

ABMs belong to the class of rule-based modeling techniques. Different frameworks for the creation and simulation of ABMs exist (Railsback et al., 2006), each with different advantages and disadvantages. We chose NetLogo (Wilensky, 1999) to conduct simulations.

In Netlogo, agents interact in a 2-dimensional world composed of microcompartments (MCs), the so-called patches. In our model, we assigned to each MC variables that could influence the agent dynamics:

- *cytokine*—this variable reflected the strength of the inflammatory signals coming from the given patch and is represented by the overall cytokine level of the patch. When high, 90% of *cytokines* could spread over the adjusting patches by releasing an equal amount of *cytokines* from the patch in every direction. *Cytokines* were released by the epithelium upon contact with *C. albicans* hypha agents. The magnitude of the *cytokine* release depended on the hyphal burden of the MC (Moyes et al., 2010). In the model we didn't distinguish between different types of cytokines. Instead, we considered all cytokines that modulate the immune response together in this *cytokines* variable;
- *nutrition*—a Boolean value indicating whether nutrient was available at the patch. On every time step an agent present on the patch could consume the nutrient if *nutrition* was 1. After being consumed *nutrition* was assigned the value 0 and it was restored with a probability of 3% in the next time step;
- *portal*—was a point of entry to the system for phagocytic cells, which became attracted to the system in a cytokine-dependent way;
- *invaded?*—the Boolean value, *invaded?* holded value TRUE for patches that were invaded by hyphae agents;
- *patch-phagocytising-energy*—a variable representing the number of *ts* needed for a PMN or a macrophage to completely phagocytise a hyphal agent or yeast agent.

**Figure 1:**
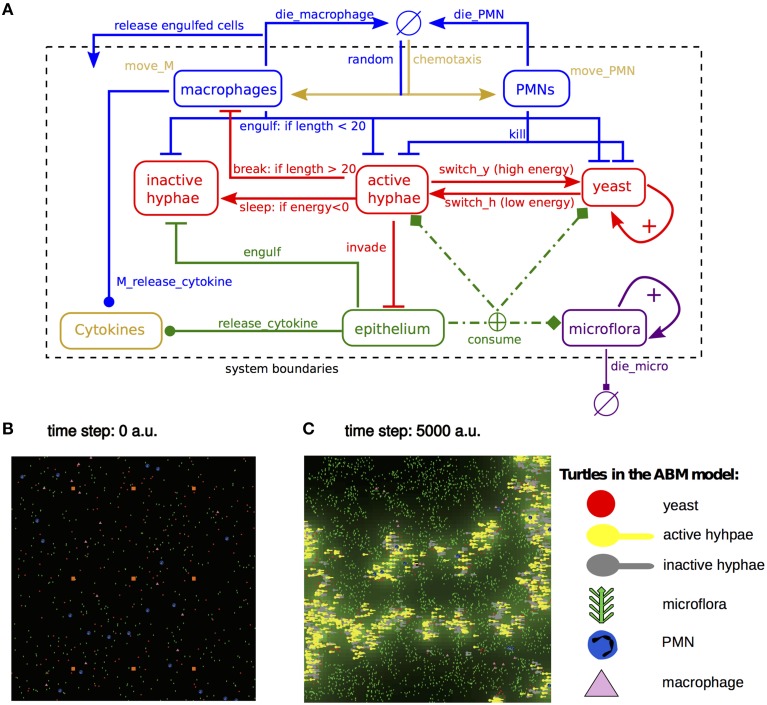
**Model description and framework. (A)** Graphical representation of the model implemented in NetLogo. Arrows indicate transitions from one agent type to another, barred lines indicate negative interactions, lines ending in circles indicate positive interactions. **(B,C)** Snapshots of the model at *t* = 0 *ts* and *t* = 5000 *ts*. *Portals*, (orange squares), provide an access for the phagocyte entering the system from the bloodstream. Agents (yeast: red; active hyphae: yellow; inactive hyphae: gray; microflora: green; PMNs: blue; macrophages: pink) are randomly spread over the world at the beginning of the simulation. At *t* = 5000 *ts C. albicans* yeast and hyphal agents as well as microflora have colonized the patches. Cytokine values are indicated by levels of green.

Our model consisted of five classes of agents: the two static *C. albicans* yeast and hyphal agents, and the three mobile agents including microflora, PMN and macrophage agents. All the interactions of agents and patches that we implemented in the model are depicted in Figure 1 and the full model code is available as Supplementary Information. In the model, *C. albicans* yeast and hypha agents, microflora, PMN and macrophage agents were randomly distributed over the world at the beginning of each simulation (Figures 1B,C).

Microflora, yeast, and active hyphae agents could consume the *nutrition* of the MC potentially at every time step, if available. Each agent had an attribute called *energy*—a variable with a numerical value, which increased upon nutrient uptake and decreased over time to reflect the metabolic state and consumption of the accumulated energy by the cells. When the agent did not consume *nutrient*, its *energy* value decreased and it could become negative when the situation prolonged. Microflora agents with a negative energy status died and were removed from the system. We set thresholds for cell division so that microflora outcompetes yeast in an unstressed scenario and stable populations can form. When the energy status of a microflora agent passed this threshold it divided and one new cell was created next to the parental cell. Similarly, every yeast agent divided when its energy status exceeded a threshold, giving rise to one parental cell and one daughter cell. Every time a yeast agent's *energy* fell below a threshold, the yeast turned into an active hyphae agent. An active hyphae agent induced inflammatory responses by increasing the *cytokine* value of the patch. An active hyphal agent could switch back to a yeast agent when it accumulated enough energy (Saville et al., 2003). Upon sparse nutrient availability, caused by a high density of other agents in the system, the energy level of an active hyphae agent eventually dropped below zero. The active hyphae agent with negative *energy* became inactive and ceased damaging epithelial cells. Inactive hyphae agents no longer increased the *cytokine* value of the patch. To prevent the accumulation of inactive hyphae in the system, MC were set to be able to engulf inactive hyphae agents, corresponding to the process of induced endocytosis (Naglik et al., 2011). Inactive hyphae could also be phagocytised by immune agents, a process we describe later on. PMN agents and macrophage agents entered the system through nine distinctive patches we named *portals* indicating access to the blood stream (Figure 1B, marked in orange, Ray et al., 2009). The rate of recruitment of both immune agent types to the system increases with the mean cytokine level over all MCs. A low basal rate was used to ensure basal immune cell recruitment even in the absence of infection. Once an immune cell had passed through the *portal*, the phagocyte moved along the *cytokine* gradient with an additional random turn between −45° and +45° at each *ts*. To prevent explosion of the system by the immune cells, PMN agents had a 3% chance per *ts* to be removed from the system. Macrophages were removed eventually through killing by hyphal cells.

Both phagocytic agents, PMNs and macrophages, were able to engulf *C. albicans* agents upon contact. The phagocytosis of the agent began right after its engulfment. If a PMN agent established contact with a *C. albicans* agent, the PMN killed the *C. albicans* agent within 4 *ts*. A PMN that was currently occupied with phagocytosis could not engulf other agents.

In contrast to PMNs, macrophages could engulf up to 80 *C. albicans* yeast and hyphal agents. Inactive hyphal agents were removed from the system by phagocytosis and they were preferentially engulfed by macrophages although PMNs could also spontaneously engulf inactive hyphal agents. Fungi engulfed in macrophages could not take up nutrients in our model and died when their energy level reached 0. Engulfed inactive hyphae did not cause harm to the macrophage and were removed from the system after phagocytosis was completed. If a macrophage agent engulfed a yeast agent, the yeast immediately turned into an active hypha within the macrophage independently of its current *energy* value. This process reflected one of the *C. albicans* immune system response evasion mechanisms: hyphal cells within a macrophage continue to grow and *C. albicans* hyphae with a certain length (set to 20 μm here) are able to escape macrophages (McKenzie et al., 2010). To account for this in the model, each hyphal agent had an additional attribute *age* corresponding to the hyphal length. Hyphal length was increased by one unit at each *ts*, independent of the *engulfed* state. To escape a macrophage, a hypha needs to grow 20 units. During the hyphal growth within the macrophage the *energy* level of the engulfed hyphae agent continued to decrease until it dropped below 0 and the agent became inactive and essentially removed from the system. However, if the hyphal agent managed to grow beyond a threshold length it would break through the macrophage agent. If this happened, any *C. albicans* agent that was inside the macrophage was immediately released to neighboring MCs (Lorenz et al., 2004). A yeast agent engulfed by a PMN agent could not turn into a hyphal agent. In the model, PMN agents always succeed in killing *C. albicans* yeasts and hyphae (Wozniok et al., 2008).

We also took into account the ability of active hyphal agents to invade epithelial tissues. To reflect this in the model, every active hyphal agent with an *age* attribute greater than 3, which means it is in a hyphal agent state for more than 3 *ts*, was able to invade a MC beneath. In the model, the complete penetration of the epithelial barrier by active hyphae agents took 10 *ts*. During this time the patch increased its *cytokine* release. We didn't model the steps afterwards, which would reflect fungal dissemination through the bloodstream.

**Figure 2:**
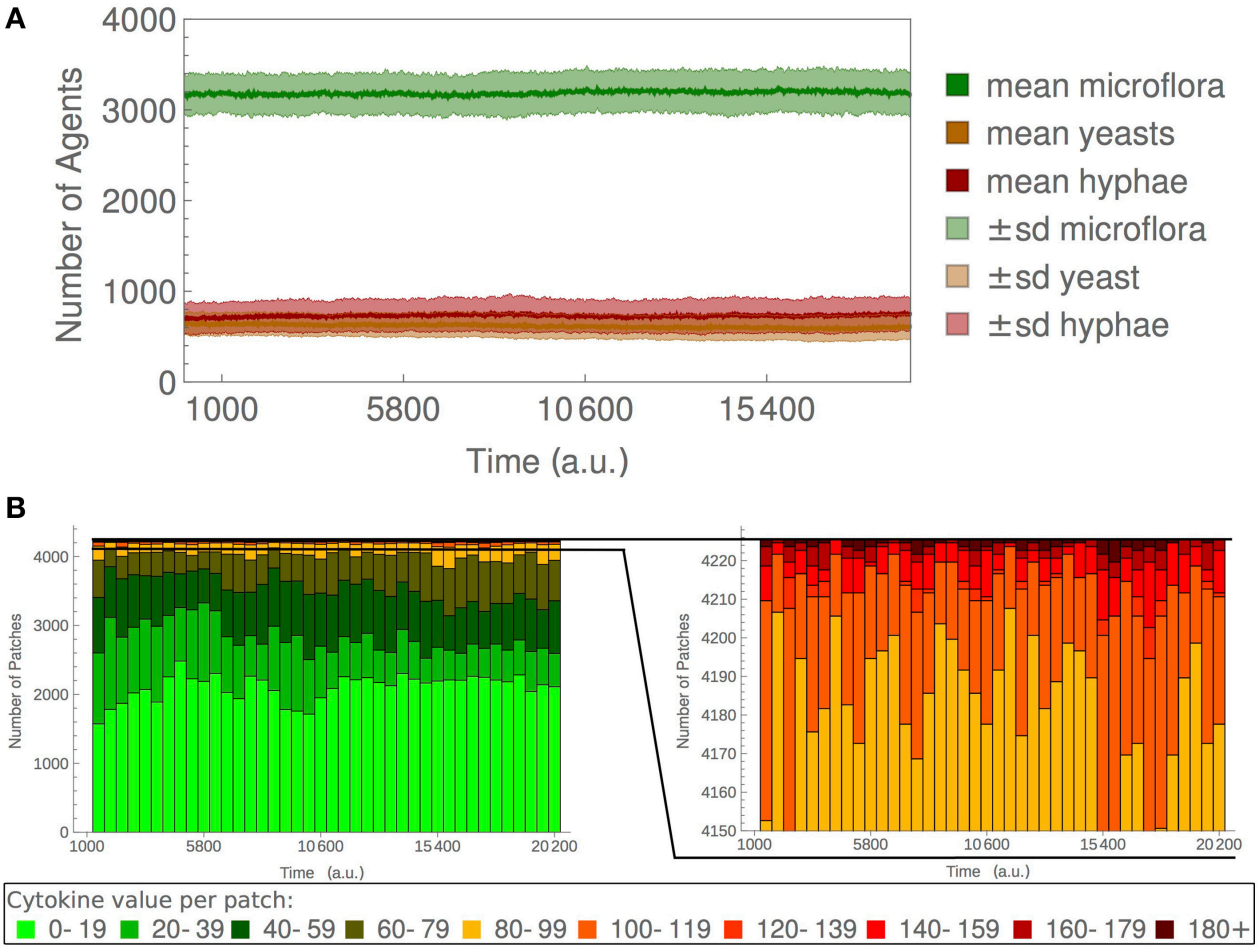
**The commensal colonization by ***C. albicans*****. The commensal state is characterized by a high amount of microflora agents, a low *C. albicans* population, and a low degree of inflammation. **(A)** Dynamics of *C. albicans* yeast, hyphae and the microflora when simulating the default model. **(B)** Number of patches with low (0–40), medium (40–80), and high (80+) cytokine values.

The parameters of the model were chosen such that microflora and fungal agents could coexist in non-stimulated conditions (Figure 2A), and such that disruption of microflora would lead to fungal overgrowth and hyphae formation, that would in turn be counteracted by activation of an immune response. The parameters are given in Supplementary Table 1.

## Scoring deviations from the reference state

To evaluate the state of the system for a given drug treatment scenario, we introduced an infection score: a score based on the number of microflora and hyphal agents in the system. It describes the relative difference between the system with intact microflora and the treated system. The score is composed of three terms:

$$\begin{array}{l} IS_{total}=\frac{1}{3}\left(IS_{microflora\text{\_}end}+IS_{hyphae\text{\_}end}+IS_{hyphae\text{\_}total}\right) \end{array}$$

The last term, “*IS*_*hyphae_total*_,” accounted for the severity of *C. albicans* infection and the inflammation over the entire course of simulation in terms of hyphae load, beginning at *t* = 0 *ts*, throughout the drug treatment, its termination and system's recovery phase. “*IS*_*hyphae_end*_” represented the state of the system in terms of hyphae load toward the end of the simulation, namely after the system has recovered or equilibrated. Similarly, “*IS*_*microflora_end*_” represented the state of the system in terms of healthy microflora toward the end of the simulation. The scores are given by the following expressions:

$$\begin{array}{l} IS_{microflora\text{\_}end}=\frac{\sum_{t=t_{start}}^{t_{end}}microflora\left(treatment,t\right)}{\sum_{t=t_{start}}^{t_{end}}microflora\left(reference,t\right)} \end{array}$$

$$\begin{array}{l} IS_{hyphae\text{\_}end}=\frac{\sum_{t=t_{start}}^{t_{end}}hyphae\left(treatment,t\right)}{\sum_{t=t_{start}}^{t_{end}}hyphae\left(reference,t\right)} \end{array}$$

$$\begin{array}{l} IS_{hyphae\text{\_}total}=\frac{\sum_{t=0}^{t_{end}}hyphae\left(treatment,t\right)}{\sum_{t=0}^{t_{end}}hyphae\left(reference,t\right)} \end{array}$$

where *t* = 0 and *t*_*end*_ indicate the start (0 *ts*) and the end of the simulation time (8000 *ts*), respectively, and *t*_*start*_ is a time point where a successful treatment is considered to have returned the system to the basal state (here: 7000 *ts*). *Agent*(*conditions,t*) indicates the number of the respective agents under the respective simulation conditions at the specified time point.

## Results

### *C. albicans* colonization of epithelial cells represented by ABM

We created the agent-based model (ABM) to analyze the population dynamics of *C. albicans* and its interactions with the host cells during the commensal colonization of the epithelium. The topology of the model is depicted in Figure 1A. The human epithelial tissue was represented in the ABM by a 2-dimensional grid made up of 4225 microcompartments (MCs). Agents that represent *C. albicans* and immune cells were randomly distributed over the grid at the beginning of every ABM simulation (Figure 1B). During the model simulation each MC regularly spawned nutrition, which was consumed by present microflora agents or *C. albicans* agents (in either yeast or hyphal form). The host immune response was incorporated in the model in the form of PMN and macrophage agents. Immune cells appeared in the system upon sensing inflammatory signals diffusing through the epithelium due to higher hyphal load. In the ABM, inflammatory signals reflected the *cytokine* release by each MC and represented the hyphal burden on the MC. Immune cells could enter the system through nine MCs, referred to as *portals* providing access to the blood stream (Ray et al., 2009) (Figure 1B, marked in orange), and move toward the infected site by sensing the cytokine gradient within the system (Figure 1C, green gradient).

The model parameters were tuned such that the microflora outcompetes *C. albicans* population during a commensal state and *C. albicans* was maintained on low levels with a predominant yeast form (Figure 2A) (Peleg et al., 2010; Cottier and Pavelka, 2012). For details, see Materials and Methods.

### Simulations of the system with intact microflora

Growth of the microflora and *C. albicans* population were limited by nutrient availability. In a commensal state, the microflora agents, which competed with *C. albicans* agents for nutrients, prevented *C. albicans* overgrowth. To simulate a healthy state we chose the number of microflora agents to be nearly twice as high as the total number of *C. albicans* agents (Figure 2A; Supplementary Table 1). In the setting with intact microflora, most MCs contained low *cytokine* levels (Figure 2B), which led to only a residual recruitment of immune cells.

Our choice of parameters allowed for the simulation of a commensal colonization by *C. albicans*, characterized by a high amount of microflora agents, a low *C. albicans* population, and a low degree of inflammation. Only low fluctuations in population sizes were observed over repeated simulations (Figure 2A), showing the robustness of the model and the reproducibility of results. We used this model as the healthy reference state of the system for all further analysis.

### Model dynamics upon disruption of microflora

**Figure 3:**
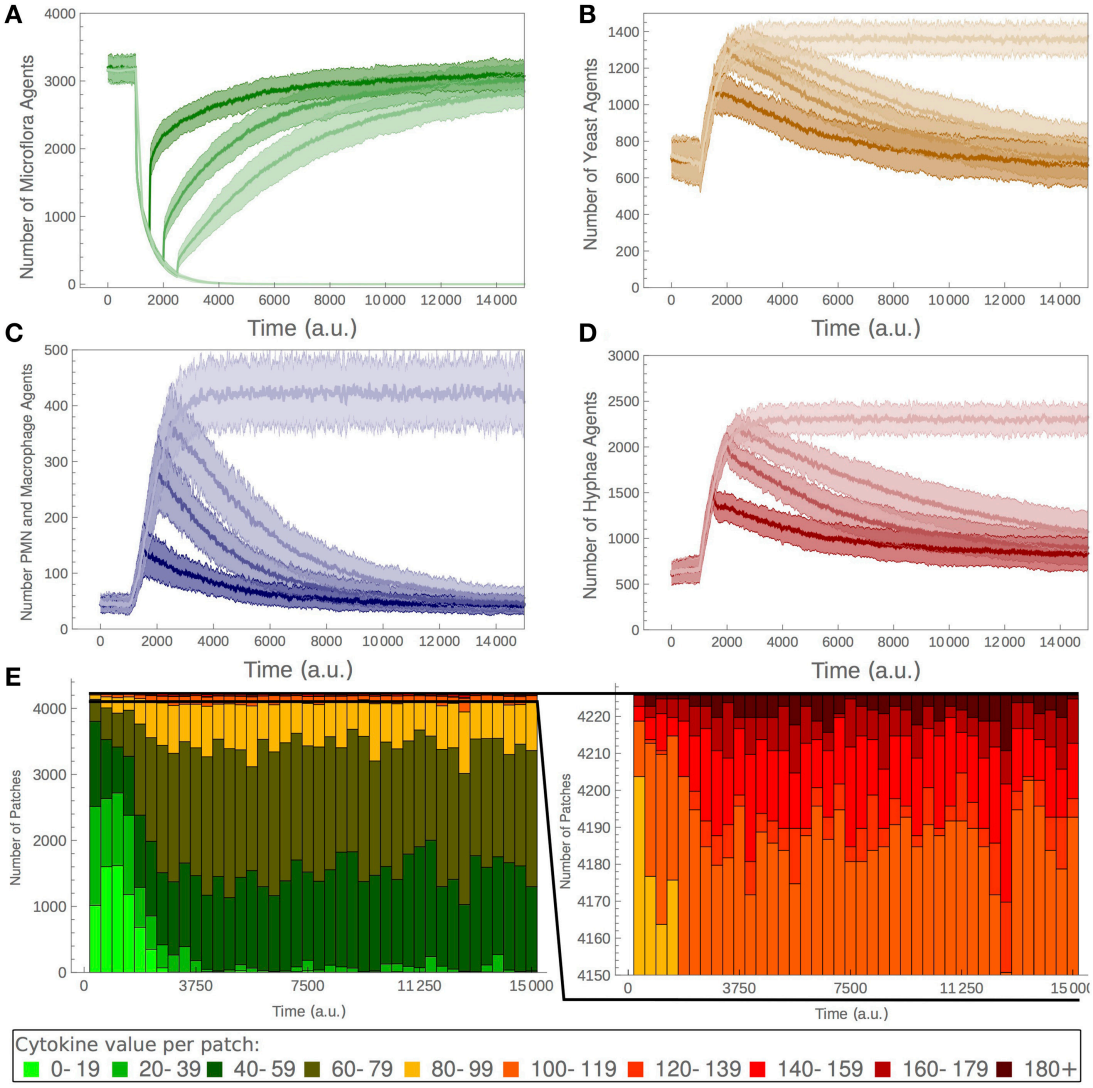
**Simulation of the model upon MD treatments**. Duration of the MD treatment (see Table 1 for more details) affects the ability of the system to recover. Model simulations with MD applied at *t* = 1000 *ts* and removed at either *t* = 1500 *ts*, 2000 *ts*, or 2500 *ts*. **(A)** Number of microflora agents, **(B)** yeast agents, **(C)** PMN and macrophage agents, and **(D)** hyphae agents. **(E)** Cytokine levels counted for all MCs upon continuous application of the MD drug.

Under certain conditions, a disruption of the microflora may result in fungal overgrowth, followed by fungal infections. Using our ABM we simulated the use of a hypothetical antibiotic that reduces the proliferation rate of the microflora agents (microflora drug; MD). We applied the MD treatment only after an initial commensal state had established, i.e., at time *t* = 1000 *ts* (Figure 3A). A constant dose of MD treatment led to a sudden decrease of the microflora population, followed by its extinction after approximately 4700 *ts* (Figure 3A). The decrease in microflora population size was accompanied by a strong increase in *C. albicans* yeast agents (Figure 3B). Saturation-like dynamics were observed due to the limited nutrient resources. The increasing fungal population resulted in nutrient depletion and a starvation signal, which subsequently induced hypha formation (Gow et al., 2012; Figure 3D). Hyphal cells in turn stimulated the cytokine release by the epithelium, leading to inflammation and phagocyte recruitment (Figure 3C). Accordingly, the distribution of patches cytokine values is shifted toward more high-cytokine patches (Figure 3E).

We analyzed the effect of different durations of the MD (summarized in Table 1). Upon termination of the treatment, each population slowly returned to the initial state (treatments ending at *t* = 1500 *ts*, 2000 *ts*, or 2500 *ts* are depicted in Figures 3A–D). The dynamic of this return was highly sensitive to the duration of drug treatment: the longer the drug application, the higher the amount of hyphal *C. albicans* agents and the slower the regeneration of the microflora. Moreover, the regeneration process showed a higher variability in the host response (Figure 3C) if the drug was applied for longer periods, indicating the instability and the lower predictability for this process for long MD treatments.

**Table 1:** **Microflora-suppressant drug treatment scenarios**.

**Microflora-suppressant drug (MD)**		****IS**_**total**_**
**treatment start (*ts*)**	**treatment end (*ts*)**	
1000	1500	0.7878
1000	2000	0.6486
1000	2500	0.5109

*Outcome of different durations of the antibiotic drug treatment on the reference model with start and end time points of the treatment. For each treatment the corresponding infection score IS_total_ was calculated. An IS_total_ close to 1 is an indicator for an intact microflora without fungal infection. The microflora's ability to recover from the treatment was highly dependent on the treatment length, reflected by the IS_total_ decrease for longer treatments*.

The presented ABM describes the early stages of epithelial infection; it does not include fungal dissemination. Although the model simulations indicated the system's ability to recover from fungal infections, one has to keep in mind that the model's time scale is arbitrary and likely surpasses the onset of fungal dissemination. Hence, we aimed to identify strategies that reduced both the duration and severity of fungal infection. We introduced an infection score to quantify the severity of the fungal infection:

$$\begin{array}{l} IS_{total}=\frac{1}{3}\left(IS_{microflora\text{\_}end}+IS_{hyphae\text{\_}end}+IS_{hyphae\text{\_}total}\right) \end{array}$$

The quantity *IS*_*total*_ combined three measures each comparing infection to a healthy reference: the amount of microflora agents at a reference interval after infection and treatment (*IS*_*microflora_end*_, did the microflora recover in comparison to the uninfected and untreated reference?), the amount of hyphae at the same reference interval (*IS*_*hyphae_end*_, was the infection cleared?), and the amount of hyphae during the whole simulation period (*IS*_*hyphae_total*_, how severe was the infection?). Since we always compared the infected and treated case to the uninfected, healthy case, an *IS*_*total*_ value of 1 indicated that the considered system has an intact microflora without *C. albicans* infection. A treatment was considered as optimal if the resulting score was reliably close to 1 depicting a successful cure of the *C. albicans* infection. A score smaller or greater than 1 indicated a disruption in the microfloral balance, having either more or less *C. albicans* agents in the treated system than in the healthy reference system, respectively (compare Figures 3A–D; Table 1). Both cases were considered unfavorable and indicated a renewed imbalance in the microflora. For details of the computation of *IS*_*total*_ see Materials and Methods.

### Model dynamics upon drug application against fungal cells

To simulate a treatment of the MD-induced infection, we applied two hypothetical drugs that did not directly kill *C. albicans* cells but either inhibited their growth (division drug; DD) or prevented the transition from yeast to hyphal state (transition drug; TD). We tested the efficiency of both drugs in clearing the fungal infection. The drugs were applied separately or in combination for different durations and dosages, after the initial onset of MD (summarized in Table 1).

#### Drug treatment 1: Inhibition of fungal growth

**Figure 4:**
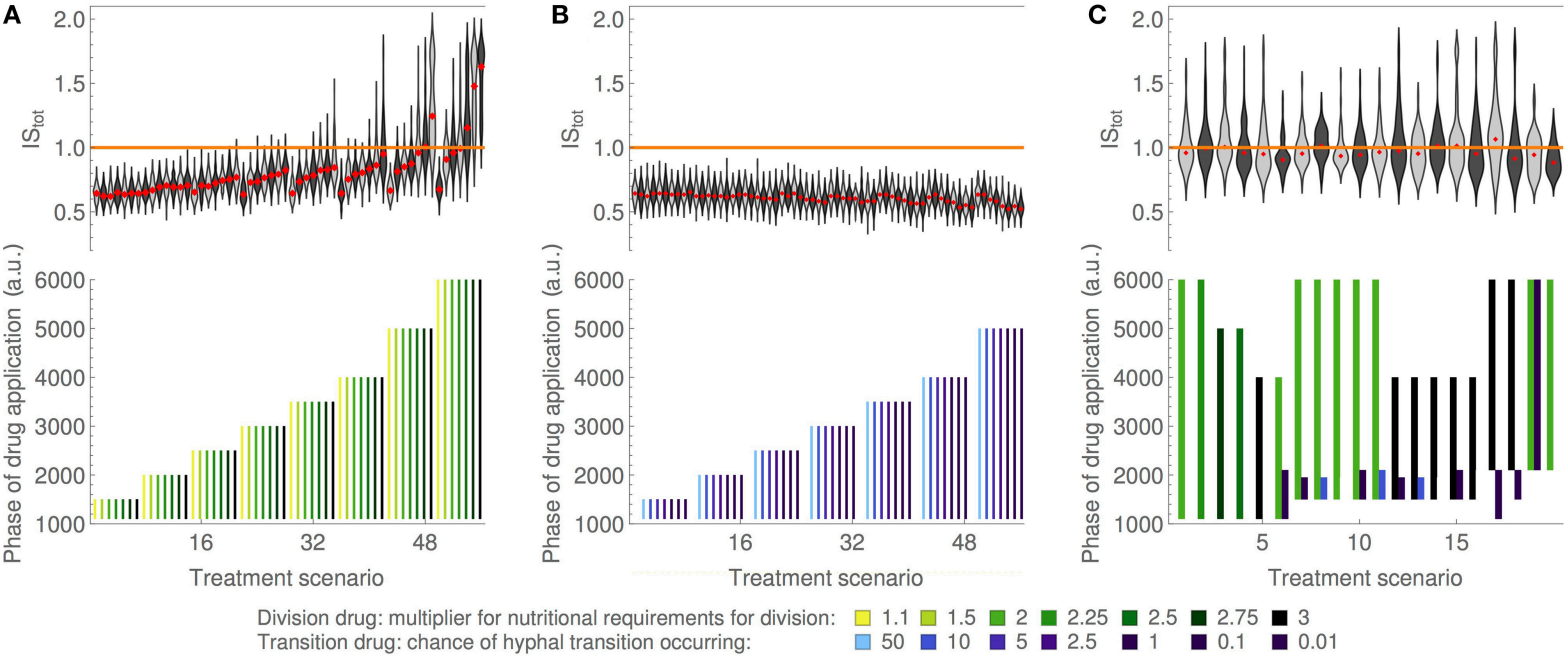
**Simulation of the model upon drug treatments**. Efficiency of drug treatment scenarios in terms of infection score. The distribution of infection scores *IS*_*total*_ is indicated by violin plots in the top panels, with the median marked in red and the optimal value of 1 indicated by a horizontal orange line. Respective treatment scenarios are indicated in the bottom panels, indicating treatment duration by bar length and dosage by bar coloring. *C. albicans* infection was always induced by the application of MD (starting at *t* = 1000 *ts*, ending at *t* = 2000 *ts*). **(A)** Analysis of the DD efficiency. Only when longer treatment was applied the system was more likely to recover from the infection by *t* = 7000 *ts*. An increased drug dosage introduced variability to the drug performance. **(B)** Analysis of the TD efficiency. Irrespective of the drug dosage and duration, the system was never able to recover from the simulated fungal infection indicating low effectiveness of the TD. **(C)** Analysis of the combinatorial DD and TD treatments. Successful treatment strategies were tested by scoring the performance of DD and TD as a multi-drug treatment. Presented are the top 20 treatments scenarios for which median *IS*_*total*_ values were close to 1 (see Table 2 for parameters). The remaining treatment scenarios tested are listed in Supplementary Table 2. Addition of TD to DD treatment has a synergistic effect. Complementation with TD decreased the *IS*_*total*_ variability and improved the median *IS*_*total*_ value when compared to the performance of DD alone with the same dose and duration (compare e.g., treatment scenarios 5 and 6, or 8 and 9).

The application of the division drug (DD) led to an increased amount of energy required for a yeast agent to divide. For instance, a DD drug dosage of 2 means that the yeast cell needed twice as much *energy* to separate into two daughter cells as under normal conditions. The DD mechanism here aimed at summarizing possible effects of fungistatic drugs that require cells to consume additional energy for adaptation and survival before division is possible, for example fluconazole (Charlier et al., 2006). Our model simulations indicated that treatment of *C. albicans* infection with DD alone could be successful if the dosage and duration were sufficient. Longer treatment durations required lower dosage, and *vice versa*, to receive an optimal *IS*_*total*_—an indicator of a successful treatment (Figure 4A). High dosages combined with high durations led to *IS*_*total*_ greater than 1 indicating that the system contained less hyphae than the healthy reference state, causing new imbalances in the microflora. The simulation variability (deviation in *IS*_*total*_ values from repeated simulations) increased as treatment duration and dosage increased (Figure 4A). Although extended treatments with higher doses tended to approach optimal *IS*_*total*_, such treatments also tended to have variable treatment outcomes (indicated by higher standard deviations in Figure 4A). This was an important result from our model simulations. It demonstrates that extended antifungal treatments with high drug doses will likely result in an unpredictable and possibly deleterious state characterized by the complete extintinction of the target—*C. albicans* in our case. Such state could lead to an increase in another microbial agent while microflora reaches a new balance. Side effects like these become highly variable when dosage and period of drugs administration are increased.

#### Drug treatment 2: Inhibition of hyphae formation

The transition drug (TD), the second drug that we tested, inhibited hyphal formation and was motivated by studies that proposed this morphogenic switch as a target for novel drugs (Jacobsen et al., 2012). In our model, a TD dosage of 1 denotes that only 1% of yeasts were able to undergo hypha formation.

**Table 2:** **Overview on the 20 best treatment scenarios as shown in Figure 4C**.

**#**	**Division drug**			**Transition drug**			**IS micro**			**IS hyphae1**			**IS hyphae2**			**IS total**		
	**Dose**	**Start**	**End**	**Dose**	**Start**	**End**	**Mean**	**Median**	***SD***	**Mean**	**Median**	***SD***	**Mean**	**Median**	***SD***	**Mean**	**Median**	***SD***
1	1.1	1100	4900	0	0	0	0.96	0.95	0.08	0.91	0.86	0.28	1.13	1.11	0.17	1	0.96	0.17
2	1.5	1100	4900	0	0	0	0.96	0.95	0.08	0.91	0.85	0.27	1.23	1.2	0.22	1.03	1	0.19
3	2	1100	3900	0	0	0	0.98	0.97	0.08	0.96	0.86	0.31	1.29	1.2	0.28	1.07	1.01	0.22
4	2.25	1100	3900	0	0	0	0.95	0.96	0.08	0.89	0.81	0.26	1.16	1.12	0.23	1	0.96	0.18
5	2.5	1100	2900	0	0	0	0.97	0.97	0.09	0.94	0.86	0.32	1.12	1.07	0.27	1.01	0.96	0.22
6	2	1100	2900	99	1100	1000	0.93	0.93	0.07	0.84	0.78	0.19	1.03	0.99	0.16	0.94	0.91	0.13
7	2	1500	4500	99	1500	450	0.95	0.95	0.07	0.87	0.82	0.21	1.15	1.14	0.15	0.99	0.96	0.14
8	2	1500	4500	90	1500	450	0.97	0.98	0.06	0.94	0.93	0.19	1.15	1.15	0.13	1.03	1.02	0.12
9	2	1500	4500	0	1500	450	0.95	0.94	0.06	0.86	0.79	0.23	1.11	1.08	0.14	0.98	0.94	0.14
10	2	1500	4500	99	1500	600	0.94	0.94	0.07	0.84	0.82	0.19	1.13	1.12	0.15	0.98	0.95	0.15
11	2	1500	4500	90	1500	600	0.95	0.95	0.07	0.9	0.84	0.25	1.14	1.12	0.18	0.99	0.97	0.16
12	3	1500	2500	99	1500	450	1	1	0.09	1.02	0.92	0.33	1.2	1.11	0.3	1.06	0.98	0.23
13	3	1500	2500	90	1500	450	0.96	0.96	0.07	0.87	0.84	0.18	1.06	1.06	0.17	0.97	0.96	0.14
14	3	1500	2500	0	1500	450	0.99	0.99	0.07	0.97	0.93	0.27	1.16	1.13	0.23	1.03	1.02	0.18
15	3	1500	2500	99	1500	600	0.99	0.98	0.11	0.99	0.87	0.37	1.21	1.15	0.31	1.06	1.02	0.26
16	3	1500	2500	0	1500	600	0.96	0.97	0.08	0.9	0.87	0.28	1.1	1.03	0.25	0.99	0.96	0.2
17	3	2100	3900	99	1100	1000	0.97	0.97	0.13	0.96	0.87	0.4	1.45	1.43	0.3	1.11	1.07	0.27
18	3	2100	3900	99	1500	600	0.92	0.9	0.12	0.83	0.7	0.37	1.23	1.15	0.29	0.99	0.92	0.25
19	2	2100	3900	99	2100	3900	0.92	0.93	0.09	0.79	0.78	0.22	1.16	1.18	0.16	0.95	0.95	0.15
20	2	2100	3900	0	2100	3900	0.95	0.94	0.07	0.85	0.81	0.19	0.95	0.95	0.11	0.91	0.89	0.12

*For all the scenarios listed, the system was challenged by MD treatment starting at t = 1000 ts, and ending at t = 2000 ts. The different treatment scenarios are listed with dosage (fold increase of nutritional division requirements for DD; percent of inhibited hyphal transition events for TD), start and end time points (in ts). We list the means, medians, and standard deviations (sd) of the respective IS_total_ values (See Equation 1). Scores close to one indicate a return of the system to the healthy reference state, small standard deviations indicate higher treatment reliability*.

Contrary to the results with a DD treatment, model simulations with different strengths and durations of TD alone showed that this treatment was less efficient than DD (Figure 4B). The best *IS*_*total*_ that could be achieved with TD treatment was 0.66, whereas an optimal *IS*_*total*_ would be close to 1. Long treatments with high TD doses led to a decrease in *IS*_*total*_ instead of the expected return of the system to the healthy state. Contrary to DD, upon TD treatment the variability across simulation repeats were consistently small regardless of the strength and duration of the treatment. Inspection of individual simulations of the model revealed that the application of TD indeed led to a lower hyphae burden during treatment. However, yeast cells accumulated during TD treatment, causing a new outbreak of fungal infection after the treatment ended. Although treatment with TD alone had to be regarded as unsuccessful in our model simulations, the stability of this treatment appeared interesting: It was independent of dose and duration unlike the DD treatment.

#### Multi-drug treatment

Although TD was not effective as a single treatment, we reasoned that its mode of action might contribute to stabilizing the efficiency of a combined treatment. To determine possible synergistic effects, we systematically screened a large number of combinatorial treatments and compared their results in terms of median *IS*_*total*_ values and their standard deviations among different simulations. Based on our initial screening of the model parameters when testing sensible drug doses and treatment durations, we decided to use three different doses for each of the two drugs and varied treatment lengths between 3500 *ts* and 6000 *ts* for DD and between 500 *ts* and 1000 *ts* for TD, also considering different treatment start times. A full list of combinatorial treatments and their associated *IS*_*total*_ values is available as Supplementary Information. Here, we focus only on those treatments that led to optimal values of *IS*_*total*_, i.e., close to 1 (Figure 4C), indicating that the treatment was successful and the treated system has renewed microflora showing no more signs of *C. albicans* infection.

Although the DD single treatments were among the best treatment scenarios tested, the addition of TD allowed for even lower DD drug dosages or shorter DD treatment durations, effectively reducing the total amount of DD that had to be administered (Figure 4C). Moreover, combinatorial treatments showed a reduced variability across repeated simulations (reflected by low deviation of associated *IS*_*total*_ values), indicating a higher predictive value of these treatments. The tested treatment scenarios contained two cases with particularly low deviations from the optimal score (treatments 6 and 9; Figure 4C). Although the median score of these two simulations is slightly lower than 1, they show very low standard deviations. Although treatment 6 requires an earlier treatment start, its duration is just about 2/3 of treatment 9 due to the synergistic effect of the two drugs in this treatment scenario.

The two drugs target different processes that altogether affect hyphae population namely by inhibiting the increase in yeast cells (DD) and inhibiting hyphae formation (TD). In our model, TD alone leads to an adverse state as yeast cells can accumulate and form hyphae upon treatment termination. The synergism emerges when TD is applied early on to prevent hyphae formation while an addition of DD to the treatment will prevent yeast cells to accumulate—a state otherwise deleterious upon the end of TD treatment. Comparison of treatment 5 with treatment 6 as well as treatment 8 with treatment 9 indicates the overall improvement in *IS*_*total*_ value for similar DD doses when TD is added.

Exemplary, we have quantified the synergy of treatment scenarios 6, 7, and 8 according to their deviation from Bliss independence (Bliss, 1939). For this quantification, we use the improvement of *IS*_*total*_ from the untreated value (see also Table 1) as effect and compare this to the treated

$$\begin{array}{l} IS_{total}:{fu}_{treatment}=\left(1-{IS}_{total}^{treatment}\right)∕\left(1-{IS}_{total}^{ref}\right). \end{array}$$

We calculate the expected additive effect as

$$\begin{array}{l} {fa}_{12}={fa}_{1}+{fa}_{2}-{fa}_{1}\cdot {fa}_{2} \end{array}$$

with *fa*_*x*_ = 1−*fu*_*x*_ and compare this to simulation results. We found that for treatment scenario 6, the effect of the combined treatment is stronger than expected from an additive model (see Table 3), whereas for scenarios 7 and 8, we observed only very slight deviations from the expected additive effect. Hence, we found that the synergistic or additive interaction of the two drugs discussed here is a matter of dose and timing.

**Table 3:** **Quantification of synergism according to Bliss independence model**.

**Scenario**	****fa**_**1**_**	****fa**_**2**_**	**${\mathrm{fa}}_{12}^{\mathrm{add}}$**	**${\mathrm{fa}}_{12}^{\mathrm{sim}}$**	**${\mathrm{fa}}_{12}^{\mathrm{sim}}\text{/}{\mathrm{fa}}_{12}^{\mathrm{add}}$**
6	0.44	−0.08	0.4	0.51	1.29
7	0.54	−0.06	0.51	0.56	1.08
8	0.56	−0.04	0.54	0.53	0.99

*For each treatment scenario tested, we combine the observed single fractional effects (fa_1_ for DD, fa_2_ for TD), computed as the relative change in IS combined to a reference scenario, the expected additive effect (${fa}_{12}^{add}$), and the simulated combined effect (${fa}_{12}^{sim}$) and the fold change between ${fa}_{12}^{add}$ and ${fa}_{12}^{sim}$*.

## Discussion

We have developed an ABM of host-pathogen interactions during *C. albicans* infection and used this to screen combinatorial drug treatments. All the drugs tested are hypothetical drugs. Nevertheless, our approach can be readily extended and refined, when data become available, and it can be used as a proxy for testing various treatment strategies. In short,

1. we provided an ABM of host-pathogen interactions ready to download and simulate;
2. we introduced a new scoring scheme as an indicator of a treatment success;
3. we described steps for screening the progress of various treatment scenarios;
4. we suggested optimal treatment strategies based on the availability of DD and TD.

### Development of an ABM of host-pathogen interactions

Based on our previous work (Tyc and Klipp, 2011), we have developed an ABM of host-pathogen interactions upon *C. albicans* infection, which efficiently described this mutual interplay during the early stages of infection. The model predicted the outcome of proposed individual and combinatorial drug treatments and enabled an initial assessment of their potential. Although our model allowed us to identify strategies to reduce initial infections, extensions that include secondary immune response and different organs to which the fungi can disseminate could yield more detailed and reliable predictions.

ABMs are well suited to investigate disease mechanisms (An et al., 2009; Su et al., 2014). Prominent are models of cell/cancer growth under different conditions and drug treatments (Bravo and Axelrod, 2013; Li et al., 2013; Wang et al., 2013; Kim et al., 2014; Su et al., 2014), host-pathogen interactions in *M. tuberculosis* granuloma (Mattila et al., 2013; Repasy et al., 2013), and details of immune response (Tokarski et al., 2012; Gong et al., 2013).

Some obstacles hamper the application of such models for quantitative predictions. Although ABMs enjoy certain popularity, there is still no well-established standard that would facilitate model reusability, exchange, and analysis (Railsback et al., 2006). Analysis of ABMs is usually computationally demanding because it relies on iterative realization of the stochastic models (Marino et al., 2008).

For the special case of host-pathogen interactions, it is challenging to generate experimental data suitable for parameterization of ABMs, but recent progress in the areas of imaging and image analysis (Mech et al., 2011) will substantially increase the amount of available data.

### Development of a scoring scheme for treatment success

A criterion to compare treatment outcomes was required to efficiently screen different treatment scenarios. Here, we introduced a scoring scheme which we named *infection score*, *IS*_*total*_, that related the changes in the amount of hyphae upon treatment when compared to the healthy reference state. Aiming for reduced treatment duration and drug dosage whilst preserving the treatment outcome, our *IS*_*total*_ allowed for a quick initial assessment of various treatment strategies. Eventually, advanced scoring schemes will need to take pharmacokinetic predictions (Thelen et al., 2011) and more details of immune activation (Gong et al., 2013), into account.

The *IS*_*total*_ value we introduced allowed detecting the effect of treatment scenarios where the patient is administered prolonged and excessive drug treatments, reflected by *IS*_*total*_ increase beyond 1. We have interpreted the standard deviation of repeated stochastic simulations as a measure for treatment reliability. Below we discuss the performance of drug combinations we found interesting with respect to their associated *IS*_*total*_ values from repeated model simulations.

### Predictions for novel therapies against *C. albicans* infection

Our results indicated that TD drugs which prevent the switch from yeast to hyphae [e.g., a farnesol-derived treatment or Allium sativum (Low et al., 2008)] can have an adverse effect, namely by leading to bursts of hyphal cells after the end of the treatment. In our model we observed a burst of hyphal cells since cells with low *energy* level that could not undergo the morphogenetic switch, accumulated, and switched to hyphae right after the treatment ended. This might be easily overlooked when not thoroughly examined with supporting model simulations. Interestingly, our simulation results indicated that addition of a TD-like drug to a DD treatment greatly improved the treatment reliability (by reducing the variability of the *IS*_*total*_ across multiple simulations) while minimizing treatment duration and drug dosage. Minimizing treatment dosage and duration reduces both treatment cost and occurrence of potential side effects. Models of host-pathogen interactions used in the context of evaluation of drug treatment strategies, here assessed by evaluating *IS*_*total*_, will be of special use especially for establishing scenarios for treating hospital-acquired fungal infections. Patients struggling with hospital-derived illnesses often suffer from a weakened immune system and are administered other drugs in parallel. With host-pathogen models combined with pharmacokinetic models, one could examine the risks of combinatorial drug treatments.

The findings of our simulations study can not be directly translated into medical practice because our model, although it represents known biological processes, is not calibrated to quantitative experimental data. Quantitative data necessary for parameterization of models like ours are not available yet. Additionally, we used hypothetical drugs that resemble mechanisms of known drugs [e.g., fluconazole for the DD drug (Charlier et al., 2006), or Allium sativum for the TD drug (Low et al., 2008)] but in our model these are not tuned to quantitatively resemble the administration of any certain dosage of existing drugs. Nevertheless, studies such as ours show cost-efficient routes to successfully exploit different mechanisms that are not successful on their own. Before model predictions can be translated into medical practice, they need to be experimentally validated. For candidiasis, prominent experimental models that could be used for testing are mouse (Kong et al., 2015) or reconstituted human epithelium (Schaller et al., 1999). As invertebrate models can be a pre-screening step before using murine models (Brunke et al., 2015), computer models can be used to reduce overall experiments.

Finally, ABMs are, as any models, limited to a certain set of interactions. Before the translation of any modeling results into medical practice, additional interactions lying outside the scope of the respective model must be carefully checked. For ABMs of host-pathogen interactions and the testing of different drug treatments as discussed here, these are possible interactions of the drugs beyond interactions on the pathogen, e.g., pharmacokinetic interactions that extend or shorten the half-life of a compound or might increase the severity of side-effects of a compound. Such interactions could be taken into account by adding input from pharmacokinetic models as input to the ABMs. Still, there might be effects between drug and the local environment that are unknown prior to their experimental observation and can thus not be included in models (Moosa et al., 2004). Accordingly, mathematical models will not abolish *in-vitro* models or animal models altogether, but they are a formidable tool to minimize animal testing and streamline the process of knowledge generation.

## Conclusions

Mathematical modeling is increasingly used for medical and pharmaceutical predictions and shows increasing rates of success (Swierniak et al., 2009; Brockmann and Helbing, 2013; Łuksza and Lässig, 2014). In this work, we presented the screening of combinatorial drug treatments in ABMs of host-pathogen interactions. A similar approach has been used to investigate synergisms in cancer treatment (Su et al., 2014). Here, we demonstrated the potential of this approach for host-pathogen systems with the prototypical example of the human pathogen *C. albicans*.

Although some drugs are not efficient on their own, our simulations demonstrated that they can greatly improve treatment success when properly combined with other drugs. By simultaneous inhibition of multiple virulence aspects of the pathogen we were able to reduce both dosage and duration, reducing the chance of off target deleterious effect. It would be very expensive—if feasible at all—to exhaustively test a sensible number of combinations experimentally to find synergistic combinations.

Although not quantitatively reliable, our results highlighted the potential of ABM approaches for clinical applications: agent-based platforms can bridge multiple temporal and/or spatial scales that occur when studying the progress of disease from host-pathogen interactions at the site of infection to the activation of the innate immune response (Bauer et al., 2009; Kirschner et al., 2014). Although such models would be very complex and their parameterization would be very demanding, a detailed model of human immune response would be highly reusable and integration with pharmacokinetic models would further boost their predictive value. The calibration of such models would require additional experimental data and, in the case of imaging data, techniques to quantitatively integrate such data.

## Funding

This work was supported by the European Commission via the SysteMTB project (contract HEALTH-2010-241587, to EK).

### Conflict of interest statement

The authors declare that the research was conducted in the absence of any commercial or financial relationships that could be construed as a potential conflict of interest.
