# Supplementary material for: Dosage and Dose Schedule Screening of Drug Combinations in Agent-Based Models Reveals Hidden Synergies
Source: Front Physiol. 2016 Jan 6;6:398. doi: 10.3389/fphys.2015.00398 (PMC4701919; doi:10.3389/fphys.2015.00398)
Supplement: Supplementary file 1 [file SupplementalMaterial.pdf]

Supplementary Material

Dosage and dose schedule screening of drug combinations in agent-based models reveals hidden synergies

Lisa C. Barros de Andrade e Sousa, Clemens Kühn, Katarzyna M. Tyc, Edda Klipp*

*** Correspondence:** Corresponding Author: edda.klipp@biologie.hu-berlin.de

Supplementary Table ST1. Parameter settings for the Agent-Based Model of *C. albicans* host-pathogen interactions

**Name**	**Parameter description**	**Value**
Model parameters:		
**energy-from-nutrient-yeast (E)**	Energy gained from nutrient consumption by a yeast cell.	20
**energy-from-nutrient-hypha**	Energy gained from nutrient consumption by a hyphal cell.	0.9 * E
**energy-from-nutrient-microflora**	Energy gained from nutrient consumption by a microflora cell.	20
**metabolism-yeast (M)**	Energy consumption of a yeast cell due to metabolic processes.	0.3
**metabolism-hypha**	Energy consumption of a hyphal cell due to metabolic processes.	0.9 * M
**metabolism-microflora**	Energy consumption of a microflora cell due to metabolic processes.	0.3
**division-threshold-yeast (DTY)**	If the energy value exceeds the threshold, the yeast cell is able to divide. The mother cell's energy is reduced by the BEY, which is assigned to the daughter cell.	2.1 * BEY
**division-threshold-microflora (DTM)**	If the energy value exceeds the threshold, the microflora cell is able to divide. The mother cell's energy is reduced by the BEM, which is assigned to the daughter cell.	2.5 * BEM

**yeast2hypha-threshold**	If the energy value of a yeast cell drops below the threshold, a yeast-to-hypha transition occurs.	0.9 * BEY
**max-length-for-phagocytosis**	The maximal hypha length during the process of phagocytosis.	20
**phagocytising-energy**	Energy gained from *C. albicans* cell engulfment by a phagocyte.	4
Stochastic elements:		
**seed-phagocytes-random**	Phagocyte recruitment independent from cytokine level.	2%
**PMN-die-random**	PMN cell death rate.	5%
**microflora-die-random**	Microflora cell death rate.	1%
Setup Parameters:		
**initial-number-yeast**	Number of yeast cells at the start of the simulation.	150
**initial-number-microflora**	Number of microflora cells at the start of the simulation.	400
**initial-number-PMNs**	Number of PMN cells at the start of the simulation.	20
**initial-number-macrophages**	Number of macrophages at the start of the simulation.	15
**birth-energy-yeast (BEY)**	Yeast cell energy assigned at its birth.	10
**birth-energy-microflora (BEM)**	Microflora cell energy assigned at its birth.	10
Drug treatment parameters:		
**microflora-suppresant drug**	A drug, which increases the division threshold for microflora cells.	x * DTY
**division drug (DD)**	A drug, which increases the division threshold for yeast cells.	x * DTM
**transition drug (TD)**	A drug, which inhibits the yeast-to-hypha transition by a specified probability.	x %

Supplementary Table ST2. Drug treatments tested.

A full overview over the drug treatments tested. Each treatment was simulated 100 times to compute mean and median scores. Microflora drug was always applied from time step 1000 to 2000. Division drug doses indicate the fold-increase in nutrition required for division under the respective treatment. Transition drug dose indicates the percentage of possible yeast to hypha transitions that occur (values greater equal 100 indicate that the drug is not active).

Division drug			Transition drug			IS micro			IS hyphae1			IS hyphae2			IS total		
dose	start	end	dose	start	end	mean	median	sd	mean	median	sd	mean	median	sd	mean	median	sd
1,1	1100	1502	0	0	0	0,82	0,83	0,06	0,62	0,62	0,09	0,49	0,49	0,03	0,65	0,65	0,06

1,5	1100	1502	0	0	0	0,81	0,81	0,06	0,6	0,58	0,08	0,48	0,48	0,04	0,63	0,63	0,06
2	1100	1502	0	0	0	0,83	0,82	0,06	0,62	0,59	0,1	0,49	0,48	0,04	0,64	0,62	0,06
2,25	1100	1502	0	0	0	0,83	0,84	0,06	0,63	0,62	0,1	0,49	0,49	0,04	0,65	0,66	0,06
2,5	1100	1502	0	0	0	0,83	0,83	0,07	0,63	0,62	0,1	0,49	0,49	0,04	0,65	0,64	0,07
2,75	1100	1502	0	0	0	0,83	0,84	0,06	0,63	0,62	0,09	0,49	0,49	0,04	0,65	0,65	0,06
3	1100	1502	0	0	0	0,83	0,85	0,06	0,63	0,62	0,09	0,49	0,49	0,03	0,65	0,65	0,06
1,1	1100	2000	0	0	0	0,84	0,84	0,06	0,64	0,61	0,09	0,51	0,51	0,04	0,66	0,66	0,06
1,5	1100	2000	0	0	0	0,85	0,84	0,07	0,65	0,64	0,1	0,54	0,53	0,05	0,68	0,68	0,07
2	1100	2000	0	0	0	0,86	0,86	0,06	0,67	0,66	0,1	0,57	0,56	0,04	0,7	0,7	0,06
2,25	1100	2000	0	0	0	0,86	0,87	0,07	0,67	0,67	0,11	0,57	0,56	0,05	0,7	0,71	0,07
2,5	1100	2000	0	0	0	0,86	0,87	0,06	0,67	0,68	0,1	0,57	0,57	0,05	0,7	0,7	0,06
2,75	1100	2000	0	0	0	0,86	0,86	0,06	0,67	0,66	0,11	0,57	0,57	0,05	0,7	0,7	0,07
3	1100	2000	0	0	0	0,87	0,88	0,06	0,68	0,67	0,09	0,58	0,58	0,05	0,71	0,71	0,06
1,1	1100	2500	0	0	0	0,83	0,83	0,07	0,63	0,6	0,1	0,51	0,51	0,04	0,66	0,66	0,07
1,5	1100	2500	0	0	0	0,86	0,87	0,07	0,68	0,69	0,11	0,59	0,59	0,05	0,71	0,71	0,07
2	1100	2500	0	0	0	0,86	0,86	0,07	0,67	0,65	0,11	0,6	0,58	0,06	0,71	0,7	0,07
2,25	1100	2500	0	0	0	0,88	0,88	0,06	0,69	0,67	0,1	0,63	0,62	0,06	0,73	0,73	0,07
2,5	1100	2500	0	0	0	0,89	0,9	0,05	0,71	0,71	0,09	0,66	0,66	0,06	0,75	0,75	0,06
2,75	1100	2500	0	0	0	0,89	0,89	0,06	0,72	0,7	0,12	0,68	0,67	0,07	0,76	0,76	0,08
3	1100	2500	0	0	0	0,9	0,9	0,06	0,74	0,72	0,12	0,7	0,7	0,08	0,78	0,78	0,08
1,1	1100	3000	0	0	0	0,82	0,81	0,07	0,62	0,59	0,1	0,51	0,51	0,04	0,65	0,64	0,07
1,5	1100	3000	0	0	0	0,87	0,88	0,07	0,69	0,71	0,11	0,63	0,63	0,06	0,73	0,74	0,07
2	1100	3000	0	0	0	0,88	0,88	0,05	0,69	0,69	0,11	0,67	0,67	0,06	0,74	0,74	0,07
2,25	1100	3000	0	0	0	0,89	0,89	0,06	0,73	0,72	0,11	0,71	0,7	0,07	0,77	0,77	0,08
2,5	1100	3000	0	0	0	0,89	0,89	0,06	0,74	0,72	0,12	0,74	0,74	0,09	0,79	0,79	0,08
2,75	1100	3000	0	0	0	0,9	0,9	0,06	0,75	0,73	0,12	0,76	0,74	0,09	0,8	0,8	0,08
3	1100	3000	0	0	0	0,91	0,92	0,06	0,77	0,77	0,12	0,8	0,8	0,09	0,83	0,83	0,08
1,1	1100	3500	0	0	0	0,82	0,82	0,06	0,62	0,61	0,1	0,52	0,52	0,04	0,65	0,65	0,06
1,5	1100	3500	0	0	0	0,87	0,87	0,06	0,69	0,69	0,1	0,66	0,65	0,06	0,74	0,74	0,07
2	1100	3500	0	0	0	0,89	0,89	0,05	0,71	0,7	0,09	0,73	0,74	0,06	0,78	0,77	0,06
2,25	1100	3500	0	0	0	0,89	0,9	0,07	0,73	0,71	0,14	0,76	0,76	0,09	0,8	0,79	0,09
2,5	1100	3500	0	0	0	0,92	0,92	0,07	0,78	0,76	0,15	0,82	0,82	0,11	0,84	0,83	0,1
2,75	1100	3500	0	0	0	0,92	0,91	0,06	0,77	0,73	0,14	0,85	0,84	0,1	0,85	0,84	0,09
3	1100	3500	0	0	0	0,93	0,93	0,06	0,82	0,77	0,19	0,92	0,87	0,16	0,89	0,85	0,13
1,1	1100	4000	0	0	0	0,83	0,82	0,08	0,63	0,61	0,12	0,52	0,52	0,04	0,66	0,65	0,08
1,5	1100	4000	0	0	0	0,89	0,89	0,06	0,72	0,7	0,13	0,7	0,7	0,07	0,77	0,76	0,08
2	1100	4000	0	0	0	0,89	0,9	0,06	0,73	0,71	0,11	0,8	0,78	0,08	0,81	0,8	0,08
2,25	1100	4000	0	0	0	0,89	0,89	0,06	0,72	0,72	0,1	0,82	0,82	0,1	0,81	0,81	0,08
2,5	1100	4000	0	0	0	0,91	0,91	0,06	0,75	0,74	0,12	0,88	0,86	0,11	0,84	0,84	0,09
2,75	1100	4000	0	0	0	0,93	0,93	0,06	0,81	0,77	0,18	0,98	0,92	0,18	0,91	0,87	0,14
3	1100	4000	0	0	0	0,97	0,97	0,09	0,94	0,86	0,32	1,12	1,07	0,27	1,01	0,96	0,22
1,1	1100	5000	0	0	0	0,84	0,84	0,06	0,64	0,63	0,09	0,54	0,54	0,04	0,67	0,67	0,06
1,5	1100	5000	0	0	0	0,92	0,92	0,06	0,77	0,73	0,14	0,81	0,81	0,09	0,83	0,82	0,09
2	1100	5000	0	0	0	0,91	0,91	0,05	0,75	0,74	0,12	0,92	0,92	0,1	0,86	0,86	0,08
2,25	1100	5000	0	0	0	0,92	0,92	0,06	0,77	0,73	0,15	0,98	0,96	0,13	0,89	0,88	0,11
2,5	1100	5000	0	0	0	0,95	0,96	0,08	0,89	0,81	0,26	1,16	1,12	0,23	1	0,96	0,18
2,75	1100	5000	0	0	0	0,98	0,97	0,08	0,96	0,86	0,31	1,29	1,2	0,28	1,07	1,01	0,22
3	1100	5000	0	0	0	1,06	1,05	0,1	1,28	1,13	0,47	1,56	1,54	0,34	1,31	1,25	0,3
1,1	1100	6000	0	0	0	0,85	0,85	0,06	0,66	0,65	0,11	0,55	0,55	0,04	0,69	0,68	0,07
1,5	1100	6000	0	0	0	0,96	0,96	0,06	0,88	0,86	0,19	0,92	0,91	0,11	0,92	0,92	0,11
2	1100	6000	0	0	0	0,96	0,95	0,08	0,91	0,86	0,28	1,13	1,11	0,17	1	0,96	0,17
2,25	1100	6000	0	0	0	0,96	0,95	0,08	0,91	0,85	0,27	1,23	1,2	0,22	1,03	1	0,19

2,5	1100	6000	0	0	0	1,02	1,02	0,09	1,16	1,01	0,43	1,5	1,46	0,3	1,23	1,16	0,27
2,75	1100	6000	0	0	0	1,09	1,1	0,08	1,48	1,52	0,45	1,76	1,86	0,27	1,44	1,48	0,27
3	1100	6000	0	0	0	1,13	1,13	0,09	1,59	1,77	0,44	1,84	2	0,22	1,52	1,63	0,24
0	0	0	101	1100	1502	0,83	0,82	0,06	0,62	0,61	0,09	0,49	0,49	0,03	0,64	0,65	0,06
0	0	0	50	1100	1502	0,81	0,81	0,08	0,6	0,6	0,1	0,49	0,48	0,04	0,63	0,64	0,07
0	0	0	10	1100	1502	0,82	0,82	0,07	0,61	0,6	0,1	0,48	0,48	0,04	0,64	0,63	0,06
0	0	0	5	1100	1502	0,83	0,82	0,06	0,62	0,61	0,09	0,49	0,49	0,04	0,65	0,65	0,06
0	0	0	2,5	1100	1502	0,83	0,83	0,07	0,63	0,62	0,11	0,49	0,49	0,04	0,65	0,65	0,07
0	0	0	1	1100	1502	0,84	0,83	0,07	0,64	0,63	0,11	0,49	0,49	0,04	0,66	0,65	0,07
0	0	0	0,1	1100	1502	0,82	0,82	0,06	0,61	0,6	0,09	0,48	0,48	0,03	0,64	0,64	0,06
0	0	0	0,01	1100	1502	0,82	0,82	0,07	0,61	0,61	0,09	0,49	0,48	0,03	0,64	0,64	0,06
0	0	0	101	1100	2000	0,83	0,83	0,06	0,63	0,61	0,1	0,49	0,49	0,04	0,65	0,64	0,06
0	0	0	50	1100	2000	0,83	0,83	0,06	0,63	0,63	0,09	0,49	0,49	0,03	0,65	0,66	0,06
0	0	0	10	1100	2000	0,82	0,81	0,07	0,61	0,6	0,1	0,49	0,49	0,04	0,64	0,63	0,06
0	0	0	5	1100	2000	0,81	0,82	0,07	0,6	0,59	0,09	0,49	0,49	0,04	0,64	0,63	0,06
0	0	0	2,5	1100	2000	0,82	0,82	0,08	0,6	0,6	0,1	0,5	0,49	0,04	0,64	0,64	0,07
0	0	0	1	1100	2000	0,81	0,82	0,07	0,6	0,58	0,09	0,5	0,5	0,04	0,64	0,63	0,06
0	0	0	0,1	1100	2000	0,8	0,81	0,07	0,6	0,58	0,08	0,5	0,5	0,04	0,63	0,63	0,06
0	0	0	0,01	1100	2000	0,81	0,8	0,07	0,59	0,58	0,1	0,5	0,5	0,04	0,63	0,62	0,07
0	0	0	101	1100	2500	0,82	0,82	0,06	0,61	0,6	0,08	0,48	0,48	0,03	0,64	0,63	0,05
0	0	0	50	1100	2500	0,82	0,82	0,06	0,62	0,62	0,09	0,49	0,49	0,03	0,64	0,64	0,06
0	0	0	10	1100	2500	0,81	0,82	0,08	0,61	0,6	0,1	0,49	0,48	0,04	0,64	0,64	0,07
0	0	0	5	1100	2500	0,81	0,82	0,07	0,59	0,6	0,08	0,49	0,49	0,03	0,63	0,63	0,06
0	0	0	2,5	1100	2500	0,79	0,78	0,07	0,58	0,56	0,1	0,5	0,49	0,04	0,62	0,62	0,07
0	0	0	1	1100	2500	0,76	0,77	0,08	0,55	0,54	0,08	0,5	0,5	0,04	0,61	0,61	0,06
0	0	0	0,1	1100	2500	0,76	0,77	0,08	0,55	0,55	0,08	0,51	0,5	0,04	0,61	0,61	0,07
0	0	0	0,01	1100	2500	0,76	0,76	0,08	0,54	0,53	0,09	0,5	0,5	0,04	0,6	0,6	0,07
0	0	0	101	1100	3000	0,83	0,83	0,06	0,61	0,61	0,08	0,49	0,49	0,03	0,65	0,65	0,06
0	0	0	50	1100	3000	0,82	0,82	0,06	0,61	0,6	0,09	0,49	0,49	0,04	0,64	0,63	0,06
0	0	0	10	1100	3000	0,82	0,83	0,07	0,62	0,61	0,1	0,5	0,51	0,04	0,64	0,65	0,07
0	0	0	5	1100	3000	0,79	0,79	0,07	0,57	0,56	0,08	0,49	0,49	0,04	0,62	0,62	0,06
0	0	0	2,5	1100	3000	0,77	0,77	0,07	0,56	0,54	0,08	0,5	0,49	0,04	0,61	0,6	0,06
0	0	0	1	1100	3000	0,75	0,75	0,07	0,53	0,51	0,07	0,52	0,52	0,04	0,6	0,6	0,06
0	0	0	0,1	1100	3000	0,74	0,74	0,08	0,53	0,52	0,08	0,52	0,52	0,04	0,6	0,59	0,07
0	0	0	0,01	1100	3000	0,73	0,74	0,07	0,52	0,51	0,07	0,52	0,52	0,04	0,58	0,58	0,06
0	0	0	101	1100	3500	0,82	0,82	0,07	0,6	0,58	0,09	0,48	0,48	0,04	0,63	0,63	0,06
0	0	0	50	1100	3500	0,82	0,81	0,07	0,61	0,59	0,1	0,49	0,48	0,04	0,64	0,63	0,07
0	0	0	10	1100	3500	0,78	0,79	0,08	0,57	0,56	0,09	0,49	0,48	0,04	0,61	0,62	0,07
0	0	0	5	1100	3500	0,77	0,78	0,07	0,55	0,55	0,07	0,49	0,49	0,03	0,61	0,61	0,06
0	0	0	2,5	1100	3500	0,76	0,77	0,07	0,54	0,55	0,06	0,51	0,51	0,03	0,6	0,61	0,05
0	0	0	1	1100	3500	0,72	0,71	0,09	0,52	0,49	0,09	0,54	0,53	0,05	0,59	0,58	0,08
0	0	0	0,1	1100	3500	0,71	0,72	0,09	0,51	0,5	0,08	0,55	0,54	0,05	0,59	0,59	0,07
0	0	0	0,01	1100	3500	0,7	0,72	0,09	0,5	0,5	0,07	0,54	0,54	0,04	0,58	0,59	0,06
0	0	0	101	1100	4000	0,83	0,83	0,06	0,61	0,6	0,08	0,49	0,49	0,03	0,64	0,64	0,05
0	0	0	50	1100	4000	0,83	0,82	0,06	0,62	0,61	0,09	0,49	0,48	0,04	0,65	0,64	0,06
0	0	0	10	1100	4000	0,8	0,79	0,07	0,59	0,58	0,09	0,49	0,49	0,04	0,63	0,62	0,06
0	0	0	5	1100	4000	0,76	0,77	0,07	0,55	0,55	0,07	0,5	0,51	0,04	0,61	0,61	0,05
0	0	0	2,5	1100	4000	0,74	0,75	0,08	0,53	0,53	0,07	0,52	0,52	0,04	0,59	0,59	0,06
0	0	0	1	1100	4000	0,67	0,68	0,09	0,48	0,48	0,06	0,56	0,56	0,04	0,57	0,57	0,06
0	0	0	0,1	1100	4000	0,67	0,68	0,09	0,48	0,47	0,07	0,56	0,55	0,05	0,57	0,57	0,06
0	0	0	0,01	1100	4000	0,67	0,69	0,09	0,48	0,48	0,06	0,56	0,56	0,04	0,57	0,57	0,06

0	0	0	101	1100	5000	0,82	0,81	0,07	0,6	0,58	0,09	0,48	0,48	0,03	0,63	0,62	0,06
0	0	0	50	1100	5000	0,81	0,81	0,07	0,6	0,6	0,09	0,49	0,49	0,04	0,64	0,64	0,06
0	0	0	10	1100	5000	0,77	0,78	0,07	0,56	0,56	0,08	0,5	0,49	0,03	0,61	0,61	0,06
0	0	0	5	1100	5000	0,73	0,74	0,09	0,52	0,51	0,08	0,51	0,51	0,04	0,59	0,59	0,07
0	0	0	2,5	1100	5000	0,69	0,7	0,08	0,5	0,5	0,06	0,54	0,54	0,04	0,58	0,58	0,06
0	0	0	1	1100	5000	0,59	0,58	0,09	0,44	0,42	0,06	0,62	0,61	0,05	0,55	0,54	0,06
0	0	0	0,1	1100	5000	0,6	0,61	0,08	0,44	0,44	0,04	0,63	0,62	0,05	0,56	0,56	0,05
0	0	0	0,01	1100	5000	0,58	0,59	0,1	0,43	0,43	0,05	0,62	0,61	0,05	0,54	0,54	0,06
0	0	0	101	1100	6000	0,82	0,83	0,07	0,62	0,61	0,09	0,48	0,48	0,04	0,64	0,64	0,06
0	0	0	50	1100	6000	0,81	0,81	0,07	0,6	0,6	0,09	0,49	0,5	0,04	0,64	0,64	0,06
0	0	0	10	1100	6000	0,75	0,75	0,06	0,54	0,53	0,07	0,51	0,51	0,03	0,6	0,6	0,05
0	0	0	5	1100	6000	0,71	0,71	0,07	0,51	0,51	0,07	0,54	0,53	0,04	0,59	0,59	0,06
0	0	0	2,5	1100	6000	0,63	0,64	0,09	0,47	0,45	0,06	0,57	0,56	0,04	0,55	0,55	0,06
0	0	0	1	1100	6000	0,48	0,49	0,09	0,41	0,4	0,03	0,71	0,7	0,05	0,53	0,53	0,05
0	0	0	0,1	1100	6000	0,51	0,51	0,08	0,41	0,4	0,04	0,72	0,71	0,06	0,55	0,55	0,06
0	0	0	0,01	1100	6000	0,49	0,5	0,08	0,41	0,4	0,03	0,71	0,7	0,05	0,53	0,53	0,05
1	2100	6000	101	1100	2100	0,83	0,83	0,06	0,63	0,62	0,09	0,49	0,48	0,03	0,65	0,65	0,06
1	2100	6000	101	1500	2100	0,81	0,81	0,06	0,59	0,58	0,08	0,48	0,47	0,04	0,62	0,62	0,05
2	2100	6000	101	1100	2100	0,92	0,92	0,06	0,79	0,76	0,19	0,91	0,9	0,12	0,87	0,85	0,12
2	2100	6000	101	1500	2100	0,92	0,92	0,08	0,79	0,74	0,19	0,91	0,88	0,12	0,87	0,85	0,12
3	2100	6000	101	1100	2100	1,16	1,19	0,1	1,74	2	0,42	1,67	1,72	0,32	1,52	1,63	0,28
3	2100	6000	101	1500	2100	1,14	1,13	0,09	1,63	1,79	0,41	1,58	1,54	0,29	1,47	1,52	0,25
1	2100	6000	10	1100	2100	0,8	0,8	0,07	0,58	0,57	0,08	0,48	0,48	0,04	0,62	0,61	0,06
1	2100	6000	10	1500	2100	0,82	0,82	0,07	0,61	0,58	0,1	0,49	0,48	0,05	0,64	0,62	0,07
2	2100	6000	10	1100	2100	0,92	0,91	0,06	0,78	0,74	0,15	0,92	0,9	0,09	0,87	0,85	0,1
2	2100	6000	10	1500	2100	0,92	0,93	0,08	0,8	0,75	0,23	0,93	0,9	0,12	0,89	0,87	0,15
3	2100	6000	10	1100	2100	1,11	1,12	0,12	1,49	1,57	0,5	1,57	1,49	0,33	1,39	1,44	0,32
3	2100	6000	10	1500	2100	1,1	1,08	0,12	1,42	1,31	0,5	1,53	1,44	0,34	1,36	1,32	0,31
1	2100	6000	1	1100	2100	0,79	0,79	0,06	0,57	0,57	0,07	0,51	0,51	0,04	0,63	0,63	0,05
1	2100	6000	1	1500	2100	0,77	0,76	0,07	0,54	0,54	0,08	0,48	0,48	0,03	0,6	0,6	0,06
2	2100	6000	1	1100	2100	0,86	0,87	0,09	0,67	0,63	0,16	0,98	0,96	0,15	0,84	0,84	0,12
2	2100	6000	1	1500	2100	0,86	0,85	0,07	0,66	0,64	0,1	0,9	0,9	0,09	0,8	0,8	0,08
3	2100	6000	1	1100	2100	0,97	0,97	0,13	0,96	0,87	0,4	1,45	1,43	0,3	1,11	1,07	0,27
3	2100	6000	1	1500	2100	0,92	0,9	0,12	0,83	0,7	0,37	1,23	1,15	0,29	0,99	0,92	0,25
1	2100	6000	101	1100	1950	0,82	0,83	0,06	0,62	0,62	0,08	0,49	0,49	0,04	0,64	0,65	0,06
1	2100	6000	101	1500	1950	0,83	0,82	0,06	0,63	0,61	0,09	0,49	0,48	0,03	0,65	0,64	0,06
2	2100	6000	101	1100	1950	0,93	0,92	0,08	0,84	0,76	0,21	0,94	0,92	0,11	0,9	0,86	0,12
2	2100	6000	101	1500	1950	0,91	0,91	0,08	0,78	0,72	0,19	0,9	0,9	0,11	0,86	0,84	0,12
3	2100	6000	101	1100	1950	1,15	1,15	0,11	1,63	2	0,47	1,6	1,6	0,34	1,46	1,58	0,29
3	2100	6000	101	1500	1950	1,13	1,13	0,11	1,61	1,77	0,45	1,56	1,49	0,32	1,45	1,53	0,28
1	2100	6000	10	1100	1950	0,81	0,82	0,07	0,6	0,6	0,09	0,49	0,49	0,04	0,64	0,64	0,06
1	2100	6000	10	1500	1950	0,82	0,82	0,06	0,61	0,59	0,09	0,49	0,48	0,04	0,64	0,63	0,06
2	2100	6000	10	1100	1950	0,93	0,92	0,07	0,82	0,78	0,21	0,94	0,93	0,14	0,89	0,87	0,13
2	2100	6000	10	1500	1950	0,93	0,93	0,06	0,81	0,78	0,16	0,94	0,94	0,09	0,89	0,89	0,1
3	2100	6000	10	1100	1950	1,17	1,18	0,1	1,74	2	0,42	1,73	1,81	0,29	1,55	1,68	0,26
3	2100	6000	10	1500	1950	1,16	1,21	0,11	1,68	2	0,44	1,7	1,85	0,34	1,52	1,69	0,29
1	2100	6000	1	1100	1950	0,82	0,83	0,07	0,62	0,6	0,1	0,53	0,51	0,05	0,66	0,65	0,07
1	2100	6000	1	1500	1950	0,79	0,79	0,07	0,58	0,56	0,1	0,49	0,49	0,04	0,62	0,62	0,07
2	2100	6000	1	1100	1950	0,9	0,91	0,07	0,75	0,73	0,16	1,04	1,03	0,13	0,89	0,89	0,11
2	2100	6000	1	1500	1950	0,92	0,91	0,06	0,76	0,73	0,14	0,97	0,96	0,1	0,88	0,87	0,09
3	2100	6000	1	1100	1950	1,09	1,12	0,15	1,45	1,65	0,56	1,72	1,97	0,36	1,44	1,6	0,35
3	2100	6000	1	1500	1950	1,12	1,15	0,12	1,56	2	0,52	1,69	1,81	0,34	1,48	1,67	0,32

1	1100	4000	101	1100	1950	0,82	0,82	0,07	0,62	0,63	0,1	0,49	0,48	0,04	0,64	0,65	0,06
1	1500	4000	101	1500	1950	0,82	0,83	0,06	0,6	0,6	0,09	0,48	0,48	0,04	0,64	0,64	0,06
2	1100	4000	101	1100	1950	0,93	0,92	0,07	0,82	0,81	0,17	0,96	0,92	0,17	0,9	0,88	0,13
2	1500	4000	101	1500	1950	0,88	0,88	0,06	0,7	0,68	0,12	0,78	0,79	0,08	0,79	0,79	0,08
3	1100	4000	101	1100	1950	1,07	1,07	0,07	1,4	1,26	0,43	1,67	1,69	0,3	1,37	1,33	0,26
3	1500	4000	101	1500	1950	0,99	0,99	0,07	0,97	0,93	0,27	1,16	1,13	0,23	1,03	1,02	0,18
1	1100	6000	101	1100	1950	0,83	0,82	0,08	0,61	0,59	0,1	0,49	0,48	0,04	0,64	0,63	0,07
1	1500	6000	101	1500	1950	0,84	0,83	0,06	0,64	0,63	0,09	0,5	0,5	0,04	0,66	0,65	0,06
2	1100	6000	101	1100	1950	1,02	1,01	0,08	1,16	1,05	0,4	1,47	1,41	0,3	1,21	1,16	0,25
2	1500	6000	101	1500	1950	0,95	0,94	0,06	0,86	0,79	0,23	1,11	1,08	0,14	0,98	0,94	0,14
3	1100	6000	101	1100	1950	1,23	1,24	0,04	1,99	2	0,06	2	2	0	1,74	1,75	0,03
3	1500	6000	101	1500	1950	1,1	1,1	0,09	1,5	1,49	0,45	1,81	1,93	0,24	1,48	1,51	0,25
1	1100	4000	10	1100	1950	0,81	0,81	0,06	0,6	0,59	0,1	0,49	0,49	0,04	0,64	0,63	0,07
1	1500	4000	10	1500	1950	0,82	0,81	0,05	0,59	0,59	0,06	0,48	0,49	0,03	0,63	0,62	0,05
2	1100	4000	10	1100	1950	0,93	0,93	0,06	0,82	0,8	0,18	0,97	0,96	0,19	0,91	0,88	0,13
2	1500	4000	10	1500	1950	0,89	0,89	0,06	0,73	0,72	0,11	0,79	0,79	0,09	0,8	0,79	0,08
3	1100	4000	10	1100	1950	1,06	1,07	0,07	1,34	1,26	0,41	1,65	1,63	0,31	1,35	1,35	0,26
3	1500	4000	10	1500	1950	0,96	0,96	0,07	0,87	0,84	0,18	1,06	1,06	0,17	0,97	0,96	0,14
1	1100	6000	10	1100	1950	0,83	0,83	0,08	0,62	0,6	0,11	0,49	0,49	0,04	0,65	0,64	0,07
1	1500	6000	10	1500	1950	0,81	0,81	0,06	0,6	0,58	0,09	0,48	0,48	0,03	0,63	0,63	0,06
2	1100	6000	10	1100	1950	1,04	1,04	0,08	1,21	1,16	0,38	1,57	1,53	0,29	1,29	1,27	0,25
2	1500	6000	10	1500	1950	0,97	0,98	0,06	0,94	0,93	0,19	1,15	1,15	0,13	1,03	1,02	0,12
3	1100	6000	10	1100	1950	1,23	1,24	0,04	1,97	2	0,09	2	2	0	1,73	1,75	0,04
3	1500	6000	10	1500	1950	1,14	1,13	0,1	1,64	1,9	0,43	1,89	2	0,22	1,56	1,67	0,23
1	1100	4000	1	1100	1950	0,81	0,8	0,07	0,59	0,58	0,08	0,52	0,51	0,04	0,64	0,63	0,06
1	1500	4000	1	1500	1950	0,79	0,8	0,08	0,58	0,59	0,09	0,49	0,5	0,04	0,62	0,62	0,06
2	1100	4000	1	1100	1950	0,93	0,93	0,06	0,82	0,78	0,16	1,03	1	0,18	0,92	0,9	0,13
2	1500	4000	1	1500	1950	0,9	0,89	0,07	0,76	0,72	0,15	0,82	0,81	0,08	0,83	0,82	0,09
3	1100	4000	1	1100	1950	1,1	1,08	0,1	1,49	1,41	0,42	1,78	1,88	0,27	1,45	1,44	0,24
3	1500	4000	1	1500	1950	1	1	0,09	1,02	0,92	0,33	1,2	1,11	0,3	1,06	0,98	0,23
1	1100	6000	1	1100	1950	0,82	0,82	0,08	0,63	0,61	0,12	0,53	0,53	0,05	0,66	0,65	0,08
1	1500	6000	1	1500	1950	0,81	0,81	0,07	0,6	0,62	0,1	0,51	0,5	0,04	0,64	0,65	0,06
2	1100	6000	1	1100	1950	1,01	1,02	0,08	1,14	1,08	0,37	1,54	1,52	0,26	1,24	1,2	0,23
2	1500	6000	1	1500	1950	0,95	0,95	0,07	0,87	0,82	0,21	1,15	1,14	0,15	0,99	0,96	0,14
3	1100	6000	1	1100	1950	1,24	1,26	0,04	2	2	0	2	2	0	1,75	1,75	0,02
3	1500	6000	1	1500	1950	1,17	1,18	0,08	1,79	2	0,34	1,96	2	0,1	1,63	1,73	0,17
1	1100	4000	101	1100	2100	0,82	0,84	0,06	0,61	0,61	0,09	0,48	0,48	0,03	0,64	0,64	0,06
1	1500	4000	101	1500	2100	0,82	0,82	0,07	0,62	0,59	0,1	0,49	0,48	0,04	0,64	0,64	0,07
2	1100	4000	101	1100	2100	0,92	0,93	0,06	0,8	0,78	0,14	0,93	0,91	0,11	0,88	0,88	0,1
2	1500	4000	101	1500	2100	0,9	0,9	0,06	0,74	0,7	0,11	0,79	0,78	0,08	0,81	0,79	0,08
3	1100	4000	101	1100	2100	1,05	1,04	0,08	1,26	1,15	0,4	1,61	1,63	0,31	1,32	1,24	0,26
3	1500	4000	101	1500	2100	0,96	0,97	0,08	0,9	0,87	0,28	1,1	1,03	0,25	0,99	0,96	0,2
1	1100	6000	101	1100	2100	0,81	0,81	0,09	0,6	0,59	0,11	0,48	0,47	0,04	0,63	0,62	0,08
1	1500	6000	101	1500	2100	0,82	0,81	0,07	0,61	0,59	0,1	0,49	0,48	0,04	0,64	0,63	0,06
2	1100	6000	101	1100	2100	1,03	1,02	0,09	1,17	1,07	0,42	1,52	1,45	0,32	1,23	1,16	0,27
2	1500	6000	101	1500	2100	0,94	0,94	0,08	0,85	0,8	0,21	1,12	1,1	0,18	0,97	0,96	0,15
3	1100	6000	101	1100	2100	1,23	1,23	0,04	1,99	2	0,05	2	2	0	1,74	1,74	0,03
3	1500	6000	101	1500	2100	1,13	1,13	0,1	1,63	1,79	0,44	1,86	2	0,23	1,53	1,6	0,25
1	1100	4000	10	1100	2100	0,8	0,81	0,08	0,59	0,57	0,11	0,49	0,48	0,05	0,62	0,62	0,07
1	1500	4000	10	1500	2100	0,82	0,82	0,06	0,61	0,62	0,09	0,49	0,49	0,03	0,64	0,64	0,06
2	1100	4000	10	1100	2100	0,92	0,92	0,05	0,8	0,79	0,15	0,93	0,91	0,12	0,88	0,88	0,1

2	1500	4000	10	1500	2100	0,91	0,91	0,06	0,76	0,75	0,11	0,8	0,79	0,09	0,82	0,81	0,08
3	1100	4000	10	1100	2100	1,06	1,06	0,08	1,32	1,25	0,4	1,66	1,65	0,31	1,34	1,33	0,25
3	1500	4000	10	1500	2100	0,98	0,98	0,07	0,95	0,88	0,26	1,15	1,12	0,24	1,02	0,98	0,18
1	1100	6000	10	1100	2100	0,82	0,81	0,07	0,6	0,58	0,1	0,49	0,48	0,04	0,63	0,62	0,07
1	1500	6000	10	1500	2100	0,83	0,83	0,07	0,63	0,61	0,1	0,5	0,49	0,04	0,65	0,65	0,06
2	1100	6000	10	1100	2100	1,02	1,03	0,07	1,13	1,06	0,31	1,52	1,54	0,24	1,22	1,21	0,19
2	1500	6000	10	1500	2100	0,95	0,95	0,07	0,9	0,84	0,25	1,14	1,12	0,18	0,99	0,97	0,16
3	1100	6000	10	1100	2100	1,21	1,22	0,06	1,92	2	0,25	1,99	2	0,03	1,71	1,74	0,11
3	1500	6000	10	1500	2100	1,14	1,14	0,08	1,66	1,99	0,4	1,89	2	0,18	1,57	1,71	0,21
1	1100	4000	1	1100	2100	0,77	0,79	0,08	0,56	0,55	0,09	0,51	0,51	0,04	0,62	0,62	0,06
1	1500	4000	1	1500	2100	0,79	0,79	0,05	0,58	0,59	0,06	0,5	0,5	0,04	0,62	0,62	0,05
2	1100	4000	1	1100	2100	0,93	0,93	0,07	0,84	0,78	0,19	1,03	0,99	0,16	0,94	0,91	0,13
2	1500	4000	1	1500	2100	0,9	0,89	0,06	0,74	0,72	0,12	0,81	0,8	0,09	0,81	0,8	0,08
3	1100	4000	1	1100	2100	1,07	1,06	0,08	1,36	1,2	0,41	1,74	1,82	0,27	1,4	1,36	0,24
3	1500	4000	1	1500	2100	0,99	0,98	0,11	0,99	0,87	0,37	1,21	1,15	0,31	1,06	1,02	0,26
1	1100	6000	1	1100	2100	0,77	0,79	0,08	0,55	0,56	0,08	0,51	0,52	0,04	0,61	0,63	0,06
1	1500	6000	1	1500	2100	0,81	0,82	0,06	0,59	0,59	0,07	0,5	0,5	0,03	0,63	0,63	0,05
2	1100	6000	1	1100	2100	1,03	1,03	0,08	1,16	1,11	0,35	1,61	1,62	0,26	1,26	1,26	0,22
2	1500	6000	1	1500	2100	0,94	0,94	0,07	0,84	0,82	0,19	1,13	1,12	0,15	0,98	0,95	0,15
3	1100	6000	1	1100	2100	1,23	1,24	0,04	1,98	2	0,13	2	2	0	1,74	1,75	0,05
3	1500	6000	1	1500	2100	1,16	1,16	0,08	1,75	2	0,36	1,93	2	0,16	1,61	1,72	0,19
1	2100	6000	101	2100	6000	0,82	0,81	0,07	0,61	0,59	0,11	0,49	0,48	0,04	0,64	0,63	0,07
2	2100	6000	101	2100	6000	0,95	0,94	0,07	0,85	0,81	0,19	0,95	0,95	0,11	0,91	0,89	0,12
3	2100	6000	101	2100	6000	1,14	1,13	0,12	1,62	2	0,48	1,59	1,55	0,34	1,46	1,58	0,3
1	2100	6000	10	2100	6000	0,74	0,73	0,08	0,53	0,52	0,08	0,5	0,5	0,03	0,59	0,57	0,06
2	2100	6000	10	2100	6000	0,86	0,86	0,07	0,68	0,66	0,13	0,91	0,89	0,12	0,83	0,8	0,12
3	2100	6000	10	2100	6000	1,15	1,18	0,11	1,66	2	0,47	1,69	1,85	0,33	1,51	1,63	0,29
1	2100	6000	1	2100	6000	0,55	0,54	0,08	0,43	0,42	0,04	0,66	0,65	0,05	0,55	0,53	0,06
2	2100	6000	1	2100	6000	0,92	0,93	0,09	0,79	0,78	0,22	1,16	1,18	0,16	0,95	0,95	0,15
3	2100	6000	1	2100	6000	1,18	1,23	0,09	1,8	2	0,39	1,83	2	0,25	1,58	1,73	0,26

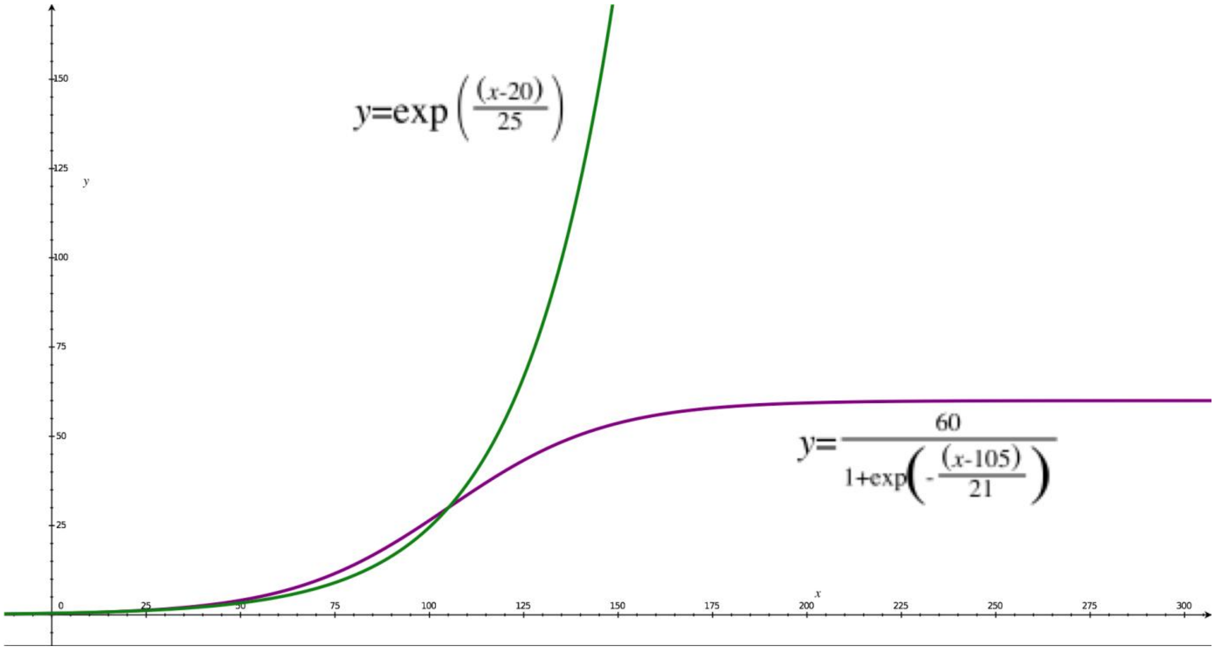

Supplementary Figure SF1. Phagocyte recruitment. The recruitment of phagocytes to the system is implemented as a function of the mean cytokine level in the simulation world. The number of PMNs or macrophages that are recruited to the system is defined by the following function (displayed in violet):

$$(1)\ x = \left\lfloor \frac{60}{1+exp\left(-\frac{mean[cytokine]-105}{21}\right)} \right\rfloor$$

The chance of recruiting phagocytes to the system is defined by another function (displayed in green):

$$(2)\ z = random(100) + \exp(\mathrm{mean[cytokine]} - 20)^{0.04}$$

$$(3)\ recruitment = \begin{cases} x \quad if\ z > (100-p)\ and\ mean[cytokine] \geq 20 \\ 1 \quad i\square\ z > (100-p)\ and\ mean[cytokine] < 20 \\ \qquad\qquad 0 \quad if\ z < (100-p) \end{cases}$$

where p is a parameter, defining the number of phagocytes that should be recruited at random.

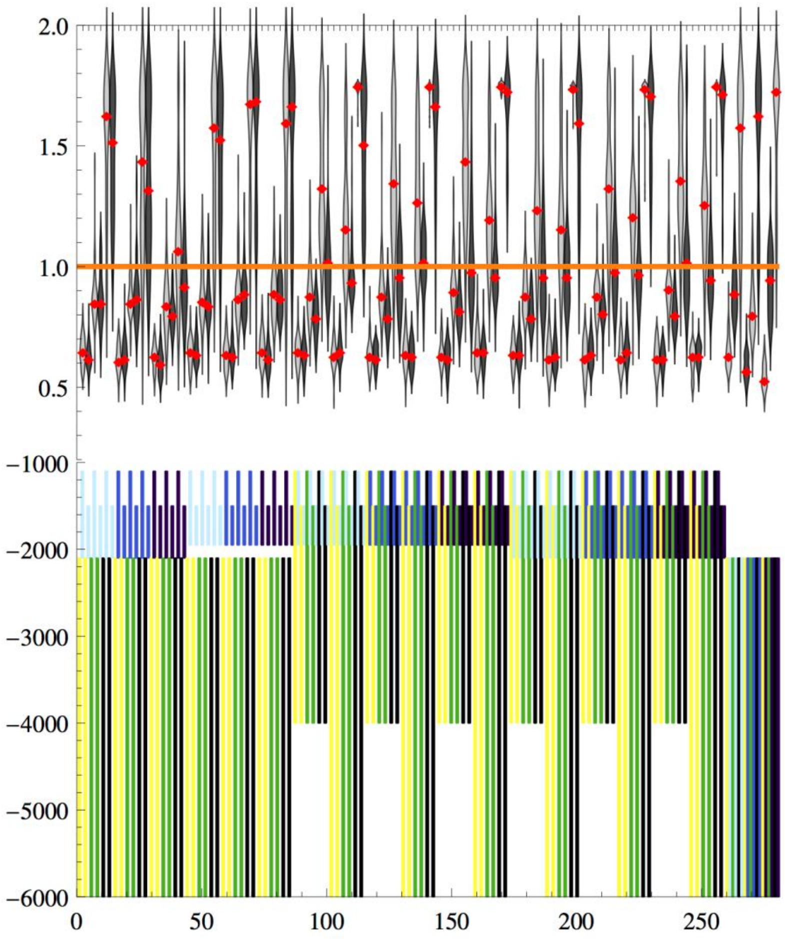

Supplementary Figure SF2. Combinatorial treatments. Analysis of the combinatorial DD and TD treatments. *C. albicans* infection was induced by the application of MD (starting at t = 1000*ts*, ending at t = 2000*ts*). Severity of the induced infection and fungal clearance was tested for different dosages and durations of TD and DD (lower panel). Each violin plot (upper panel) represents the distribution of the calculated IS_{total} values across 100 simulations for one treatment scenario. The red dot indicates the median value among 100 calculated IS_{total} values. The orange line marks the optimal score value of 1, indicating the recovery of the microflora and a successful treat
